# Advances in research on deformation and recrystallization for the development of high-functional steels

**DOI:** 10.1080/14686996.2019.1710013

**Published:** 2020-01-24

**Authors:** Kohsaku Ushioda

**Affiliations:** Research Center for Structural Materials, National Institute for Materials Science, Tsukuba, Japan

**Keywords:** Deformation, recrystallization, microstructure, texture, steel, analytical technology, computational materials science, 106 Metallic materials, 302 Crystallization / Heat treatment / Crystal growth, 303 Mechanical / Physical processing

## Abstract

In order to exploit full potential of materials, it is necessary to fundamentally control their microstructures through grain refinement and orientation. Deformation and recrystallization are means to control the microstructure. Some recent examples of research on deformation and recrystallization which make use of advanced analytical techniques and computational materials science are examined and current limitations are identified. Finally, the potential for future developments is considered with respect to the unresolved technical problems that must be addressed as part of the development of new steels.

## Graphical Abstract

**Figure:**
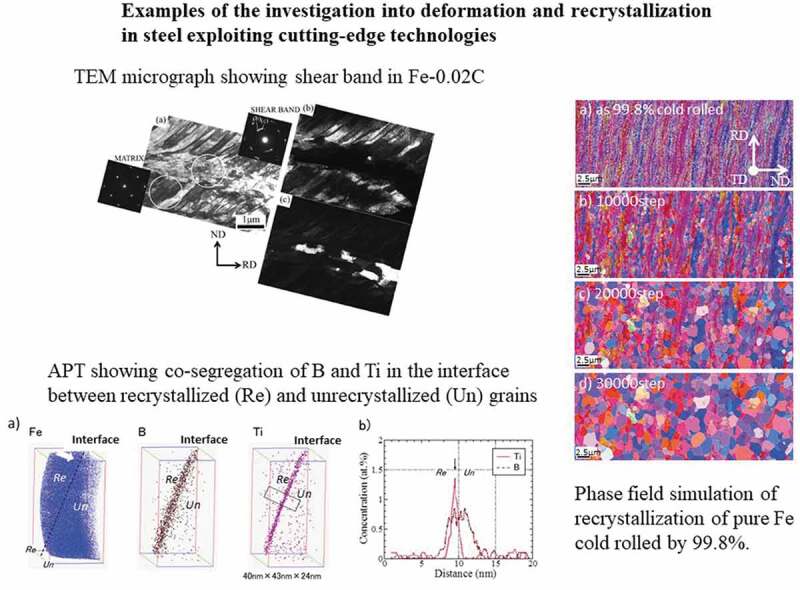

## 1. Introduction

The potential strength of commercially used metallic materials has only been partially realized. Therefore, further research is required to increase their strength [1,2]. At the same time, it is also necessary to preserve properties such as formability and fracture resistance of structural metallic materials, which are vital for their reliability. Grain refinement is one of the most effective and fundamental techniques that can be used to meet these demands. Ultra-steel research, which aimed to achieve an average ferrite (α) grain size of 1 μm [3], and research into bulk nano-metallic materials, which aimed to obtain an average grain size of less than 1 μm [4], are typical of the research currently being performed in Japan. Recently it has been reported that high carbon steel consisting of fine martensite (αʹ) transformed from fine austenite (γ) with a grain size of ~4 μm exhibits excellent ductility and fracture toughness balanced with extremely high strength greater than 2.4 GPa [5]. Moreover, Torizuka recently proposed the 3rd generation of high strength-good performance ultra-steel with a fine martensitic structure consisting of a single variant transformed from very fine austenite (γ) grains with ~2 μm in diameter [6]. Improvements in the strength-ductility balance due to grain refinement have also been reported for other metallic materials such as Ti-6Al-4V (mass%) [7] and Al alloys [8]. It should be also mentioned that grain refinement may basically improve the strength-toughness balance.

The controls of deformation and recrystallization are important as a way to refine grains but also as a means to control the crystal orientation (texture of polycrystalline metals). For example, texture controls are used in automotive panels as well as the motors of hybrid or electric vehicles, and they contribute to the high performance of the final products.

The thermo-mechanical controlled process (TMCP) is the basic concept underlying grain refinement. A combination of controlled rolling and controlled cooling is used to produce refined α grains through (1) γ recrystallization/non-recrystallization and (2) γ to α phase transformation. Since the recrystallization and phase transformation occur heterogeneously, local variations in strain and orientation are essential. The basic method for controlling the texture is as follows. Based on the generally accepted orientated nucleation and selected growth theory, both the heterogeneous deformation structure and deformed specific structure are crucial. The heterogeneous deformation structure is required for the recrystallized grains to nucleate with a preferred orientation and the deformed specific structure is required for the nuclei to grow in a selective manner. Consequently, it is necessary to understand and control the heterogeneity of the deformation structure in order to achieve grain refinement and control the texture. However, due to their complexity the technology required to evaluate and predict heterogeneous deformation structures has not yet been fully established. Therefore, rigorous microstructure controls exploiting cutting-edge technologies are anticipated.

In this overview, recent research into deformation and recrystallization using advanced novel techniques and computational materials science are introduced. In addition, potential future developments are discussed.

## 2. Research into deformation and recrystallization exploiting cutting-edge techniques

### 2.1. Heterogeneous deformation structure

The deformation structure of polycrystal metallic materials after rolling is extremely complicated. In particular, the area surrounding the initial grain boundary is heterogeneously deformed due to the constraints from neighboring grains. In addition, the heterogeneous structure in the grain interior includes microbands, shear bands, and transition bands depending on the initial orientation, grain size, chemical composition, reduction rate etc. Moreover, a locally heterogeneous structure develops at the interface between the matrix and the hard second phase. Thus, attention should be given to heterogeneous structures such as the initial grain boundary, shear bands, and the interface between the soft matrix and the hard second phase.

#### 2.1.1. Initial grain boundary region

**Figure 1.**
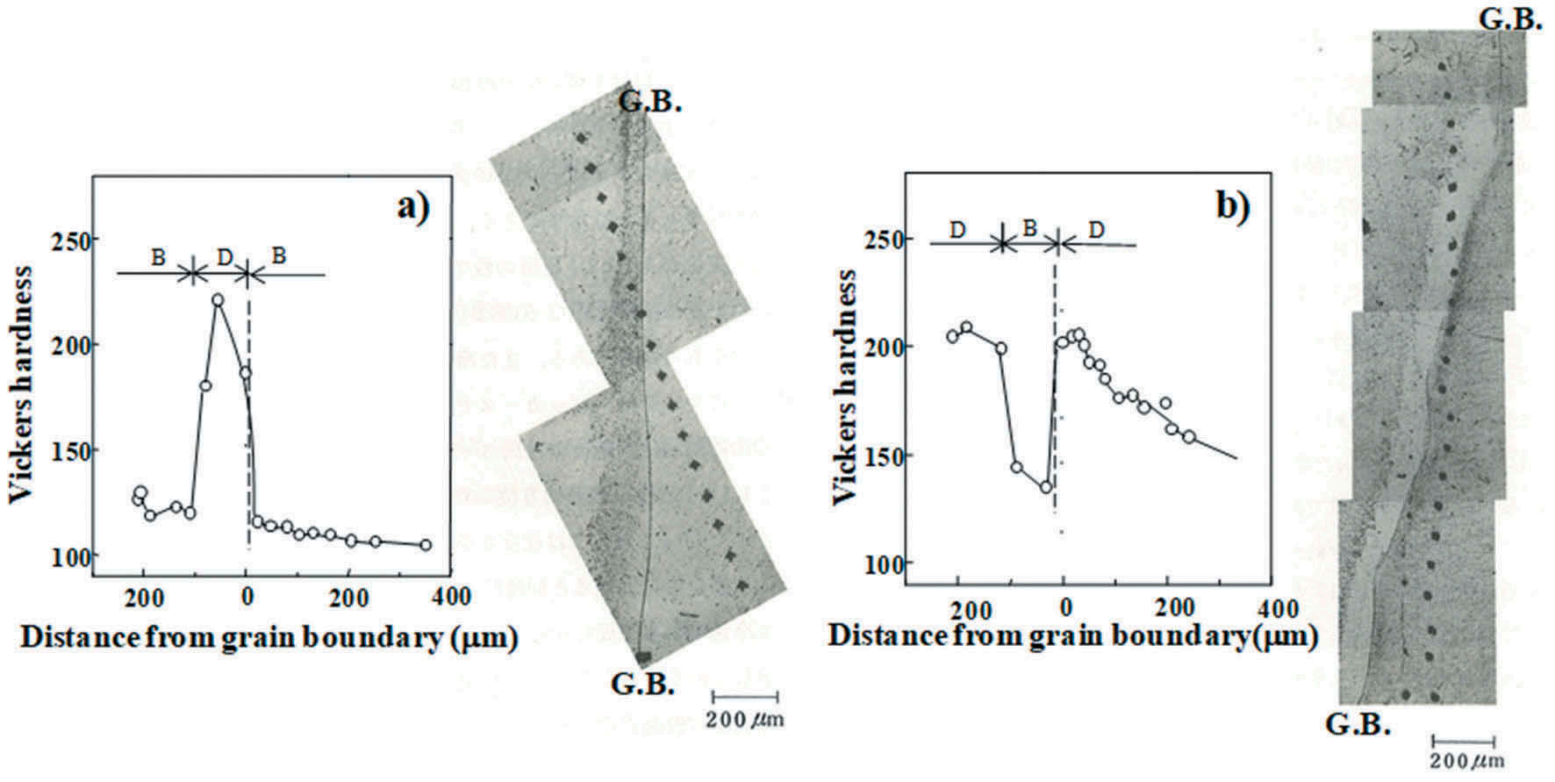
Optical micrographs showing the heterogeneous structure in the vicinity of the initial grain boundary of 70% cold-rolled pure Fe (ND plane) together with changes in the micro Vickers hardness as a function of distance from the grain boundary; (a) hardened and (b) softened regions [9]. G.B. is grain boundary. With permission of Japan Inst. Met. Mater. Reproduced by permission from [9], copyright [1982, the Japan Inst. Met. Mater.]

**Figure 2.**
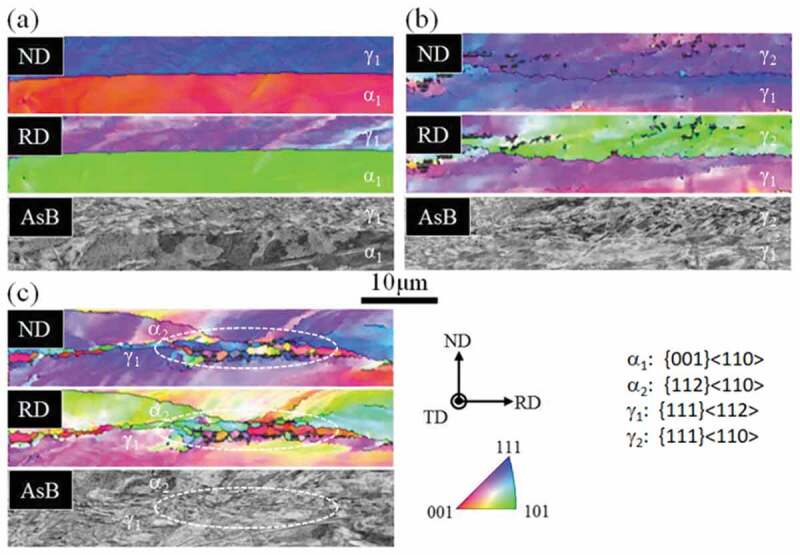
Morphological classification of the grain boundaries of 70% cold-rolled Ti-added ultra low-carbon steel; (a) relatively flat boundary, (b) irregularly serrated boundary, and (c) boundary associated with fine grains [10]. With permission of the Iron and Steel Institute of Japan(ISIJ). Reproduced by permission from [10], copyright [2017, the Iron and Steel Institute of Japan (ISIJ)]

**Figure 3.**
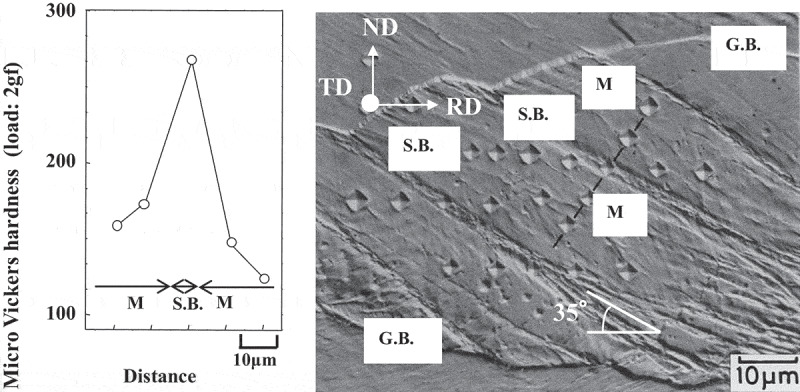
Optical micrograph showing a shear band in Fe-0.01N, 50% warm rolled under dynamic strain aging condition together with the micro Vickers hardness [11]. S.B.: shear band, M: matrix, and G.B.: grain boundary. With permission of ISIJ. Reproduced by permission from [11], copyright [1984, ISIJ]

Abe at el. investigated heterogeneity in the deformation structure focusing on the initial grain boundary in pure polycrystalline Fe after it was cold rolled by 70% [9]. In general, the grain boundary region became harder (Figure 1(a)); however, they also reported a case where the grain boundary region became softer (Figure 1(b)). The hardening behavior is believed to occur because multiple slips are required to achieve the compatibility condition which results in a locally high dislocation density. However, the less frequent softening behavior is interesting and there is currently no systematic understanding of the difference in heterogeneity in terms of the grain boundary characteristics. According to a recent study using electron backscatter diffraction (EBSD) technique [10], the heterogeneity in the vicinity of the initial grain boundary of Ti-interstitial free (IF) steel may be classified into three types: (1) relatively flat boundary (Figure 2(a), (2) irregularly serrated boundary (Figure 2(b)), and (3) boundary associated with fine grains (Figure 3(c)). Statistical analysis using EBSD and a scanning electron microscope (SEM) with angle selective backscattered electrons (AsB) was conducted for 84 types of grain boundary. The results suggest that the heterogeneity of the deformation structure in the vicinity of a grain boundary is a result of differences in the plastic deformation of the ND//<111> (γ fiber) grains and the RD//<011> (α fiber) grains; where ND and RD are the normal and rolling directions, respectively. Moreover, the existence of very fine grains frequently with ND//<111> in the grain boundary regions was also observed. This is believed to be caused by the concentration of strain at the initial grain boundary followed by dynamic recovery, which even occurs at room temperature. However, no clear explanation has been proposed for the softening hardness trend observed in the vicinity of the initial grain boundary. Therefore, further research is required including experiments using advanced technology and simulations based on crystal plasticity.

#### 2.1.2. Shear bands

**Figure 4.**
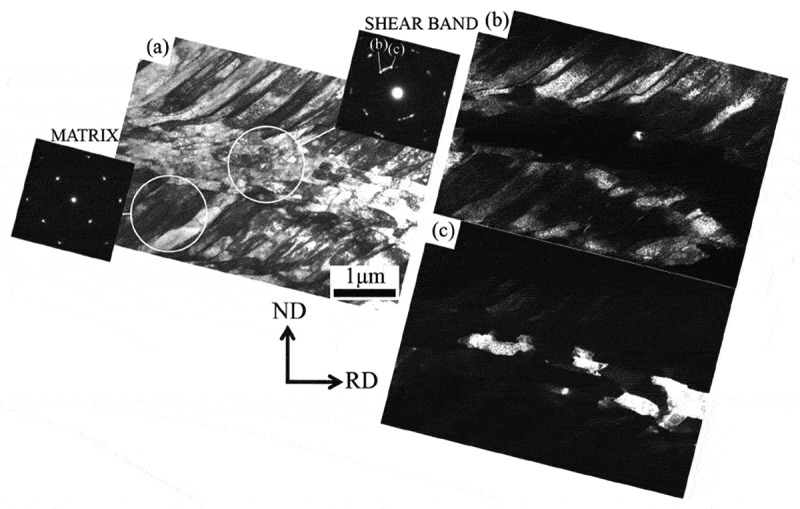
TEM images and diffraction patterns obtained from shear band and matrix after 70% cold-rolling Fe-0.02C with C in solid solution; (a) bright-field image, (b) and (c) dark-field images using the diffraction spots indicated in the diffraction pattern obtained from the shear band [12]. With permission of ISIJ. Reproduced by permission from [12], copyright [2012, ISIJ]

Small local fluctuations in crystal orientation within grains are generally expected after rolling. Furthermore, heterogeneously deformed regions occur within grains where there is immense shear strain; for example, shear bands are associated with significant crystal rotation. Figure 3 shows a typical example where a clear shear band is formed within the {111}<112> matrix when a polycrystalline Fe-0.01N alloy is rolled under dynamic strain aging (DSA) condition [11]. Similar shear bands are frequently observed after cold/warm rolling of steel with C in solid solution due to DSA [12–14]. Figure 4 shows a typical TEM image of a shear band including the microstructure and orientation within the shear band after 70% cold rolling a specimen of Fe-0.02C with C in solid solution at room temperature [12]. It is well known that rolling a single crystal of Fe-3Si with (111)[11-2] orientation will form shear bands [15,16]. This orientation has a high Taylor factor, which leads to plastic instability, as in the case of rolling under DSA condition, which results in the formation of shear bands. Recent investigations into the microstructure within shear bands by means of a transmission electron microscope (TEM) and EBSD have revealed that crystal rotation occurs locally from (111)[11-2] to (110)[001] along TD// [1-10]. This is due to the introduction of immense shear which results in very fine equi-axed cells comprised of locally rearranged dislocations with rather low density [12,14,16]. It is noteworthy that recrystallized grains with {110}<001> orientation preferentially nucleate in shear bands [11–16].

Recently another type of shear band is reported by Nguyen-Minh et al. [17] when material having {110}<110> oriented grain is rolled, which contribute to the nucleation of {100}<001> recrystallized grain. Shear band within a grain is expected to form, because {110}<110> oriented grain is a grain having very high Taylor factor.

The transition band parallel to the rolling direction is another example of heterogeneous deformation caused by rolling material with initial {100}<001> orientation [18]. The transition band is interpreted as a locally banded region where the initial orientation remains {100}<001>, while neighboring regions gradually rotate to the stable orientation {100}<011> rotated 45° along the ND//<100> axis in the alternatively opposite direction. The transition band played an important role as the preferential nucleation site of cube recrystallized grains [18]. However, the role of transition band has not been fully clarified yet. Therefore, the detailed reinvestigation into transition band seems to be required by exploiting recent advanced technologies. As for the nucleation of recrystallized grains in the grain interior, deformation twins, which frequently form after cold rolling Fe-3Si alloy with 0.018C, reportedly act as nucleation sites for recrystallized grains with {411}<148> orientation [19]. The role of deformation twin should be thoroughly investigated in future, because {411}<148> recrystallized grain may form without deformation twin.

#### 2.1.3. Hard second phase

**Figure 5.**
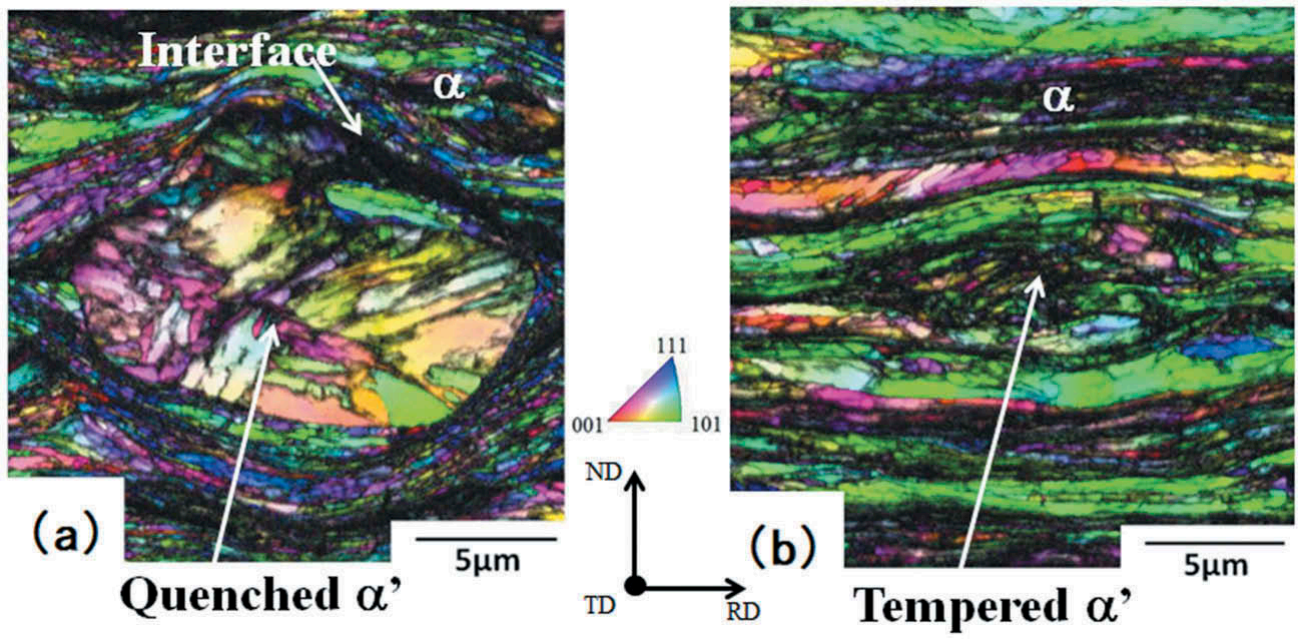
Inverse pole figure maps with respect to RD focusing on martensite after 75% cold-rolling. Specimens were (a) quenched after intercritical annealing of Fe-17Cr and (b) subsequently tempered at 550°C for 1 h before cold-rolling. The hardness ratios *k* (Hv_αʹ_/Hv_α_ prior to cold-rolling) in (a) and (b) are 2.11 and 1.70, respectively [20]

The presence of a hard second phase in the relatively soft matrix generally leads to locally complex severe deformation at the interface. Kimura et al. [20] prepared a specimen consisting of soft α and hard αʹ using an Fe-17Cr alloy by intercritical annealing followed by quenching and subsequent cold-rolling by 75%. Figure 5(a) shows the locally concentrated large strain at the interface between α and αʹ. The kernel average misorientation (KAM), which is assumed to represent the density of geometrically necessary (GN) dislocations as reported by Umezaki et al. [21], is confirmed to be locally high at the interface. However, it should be mentioned that the connection between KAM and density of GN dislocations is complicated. In contrast, the strain concentration at the interface can be reduced by softening αʹ by temper treatment prior to cold rolling (Figure 5(b)). When the hardness ratio $Hv_{\alpha^{\prime }}/Hv_{\alpha }$ is greater than 2.0, the deformation of αʹ becomes relatively difficult which results in the extremely high concentration of strain at the interface. Moreover, randomly oriented recrystallized grains nucleate in such regions, which destroys the colony structure with RD//<011> and improves the surface quality by reducing the ridging patterns [20].

### 2.2. Reconstruction of prior austenite structure from martensite by means of EBSD and its application to deformation/recrystallization behavior of austenite

**Figure 6.**
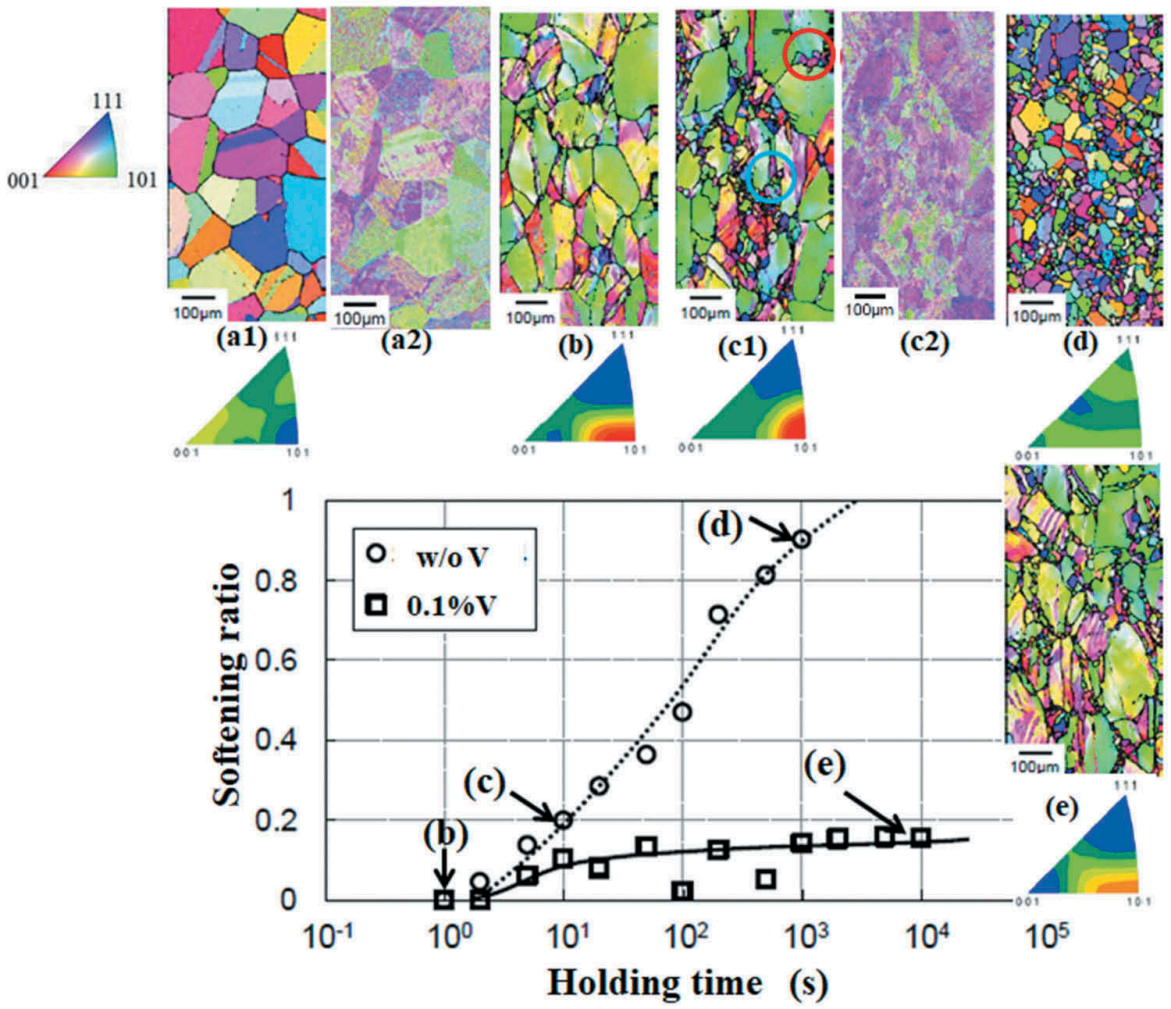
Static softening behavior of γ in 0.55C steel without and with V compressed (ε = 0.3) at 800°C together with inverse pole figure maps (CD) and inverse pole figures of γ reconstructed from martensite [24]. (a) Before the compression test and (b–e) after the compression test. CD (compression direction) is the horizontal direction

It is necessary to accurately evaluate the deformation and recrystallization behavior of γ in order to pursue grain refinement. However, it is difficult to directly observe the microstructure of γ in steels, because γ to α phase transformation takes place. Miyamoto et al. have developed a technique where the γ structure can be reconstructed from EBSD data of αʹ quenched from the γ phase [22]. This makes it possible to analyze the orientation of γ grains as well as their deformation and recrystallization behavior. An example of this is shown in Figure 6 [23]. The 0.55C-1.5Si-0.7Mn-0.7Cr steels with and without the addition of 0.1V were heated to 1200°C then rapidly cooled to 800°C (γ phase). Next, they were isothermally compressed, with strain ε = 0.3 and strain rate 2.5 s^−1^, as the first deformation. They were then held at 800°C until the second compression test with *ε *= 0.3 at the same temperature and strain rate. The αʹ microstructure was observed after quenching specimens immediately before the first and second compression tests. Figure 6 shows the change in the softening ratio ($X=\sigma_{\varepsilon }-\sigma_{y2}/\sigma_{\varepsilon }-\sigma_{y1}$) as a function of holding time after the first compression test at 800°C. Here, *σ_y1_, σ_y2_*, and *σ_ε_* represent the yield stress during the first and second compression tests, and the flow stress at *ε *= 0.3 during the first compression test, respectively. Immediately before the first and second compression tests, the samples were quenched and subjected to EBSD observation. As shown in Figure 6, the reconstruction method proposed by Miyamoto et al. [22] enables us to obtain very clear reconstructed images of the γ structure from the indistinct EBSD data for the αʹ structure. The microstructure of the γ grains before the first compression test consisted of equi-axed grains with a diameter of approximately 100 μm and annealing twins (Figure 6(a)) (1). The elongated deformed γ structure that was present immediately after the compression test can be observed in Figure 6(b). In the steel without V, softening began immediately after the compressive deformation, the nucleation of recrystallized grains occurred at both the γ grain boundaries and annealing twins after 10 s at 800°C (red and blue circles in Figure 6(c1), respectively). Recrystallization was completed after 10^3^ s (Figure 6(d)). The EBSD image for αʹ, shown in Figure 6(c2), is too indistinct to assess the recrystallization behavior of γ. In the steel with the 0.1V addition, softening was significantly suppressed and the material still remained in the early stage of recrystallization even after it was held at 800°C for 10^4^ s (Figure 6(e)). In addition, recrystallized grains also nucleated at the γ grain boundaries and annealing twins were observed. It should be noted that this method makes it possible to also obtain information regarding the crystal orientations of the γ phase. The inverse pole figures shown in Figure 6 demonstrate that the unrecrystallized γ had a strong CD//<011> texture, while that of recrystallized γ grains was weakened and randomized (Figure 6). Here, CD is the compression direction. The details of the mechanism by which the addition of V retards the recovery and recrystallization are still unclear; however, it is believed to be caused by clusters of the substitutional element and C [23,24]. It is well known that microalloying elements such as V retard γ recrystallization resulting in grain refinement of the transformed α microstructure. However, our understanding of the accurate mechanism underlying the effect of alloying elements is still insufficient and further research is required.

### 2.3. Segregation of solute elements at the interface between recrystallized/unrecrystallized grains and its effect on interface velocity

**Figure 7.**
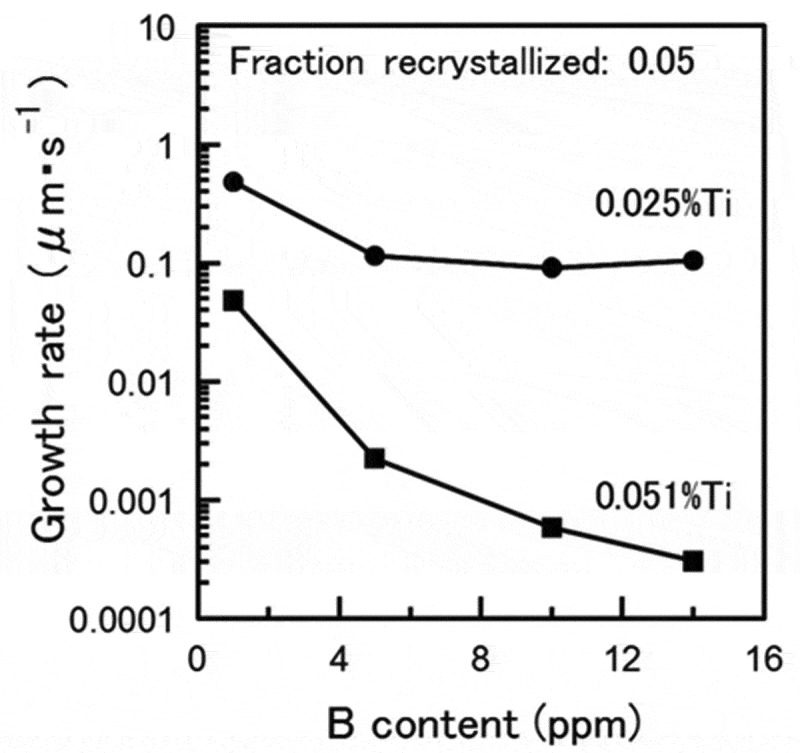
Effect of B content on the growth rate of recrystallized grains at 5% fraction recrystallized in Ti-IF steels [26]. With permission of ISIJ. Reproduced by permission from [26], copyright [2017, ISIJ]

**Figure 8.**
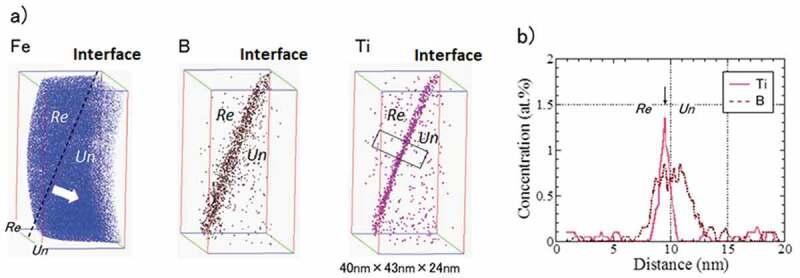
(a) 3D elemental maps and (b) concentration profiles for Ti and B across the interface between recrystallized and unrecrystallized grains in 0.0018C-0.051Ti-0.0014B steel annealed at 650°C for 1 h [25]. Arrow shows growth direction of interface. (Re: recrystallized grain, Un: unrecrystallized grain)

**Figure 9.**
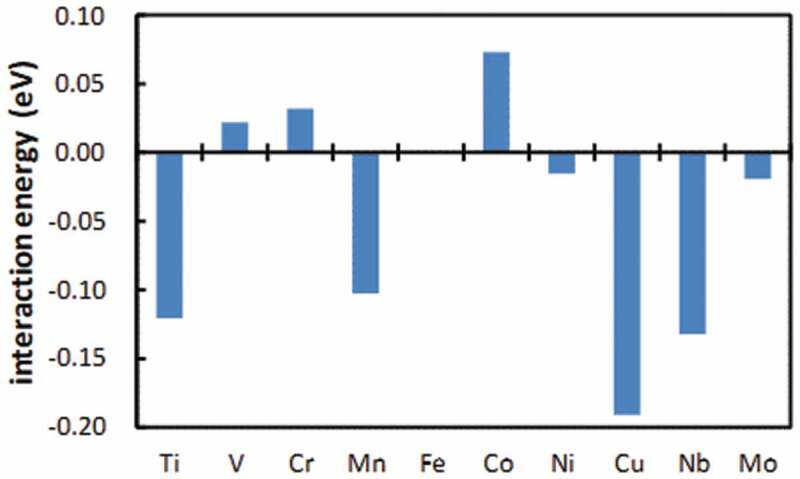
First principle calculated interaction energy between the transition metal element M and the B atom in α-Fe [27]. With permission of Nippon Steel Corp. Reproduced by permission from [27], copyright [2018, Nippon Steel Corp.]

In order to optimize the microstructure according to the required properties, it is necessary to control the velocity of the interface between recrystallized and unrecrystallized grains. The growth of recrystallized nuclei into the unrecrystallized region is known to be inhibited both by the solute drag effect due to the segregated solute atoms present in the interface, and by the pinning effect due to the presence of pinning particles in the interface. However, it has been difficult to determine the actual state of the alloying elements in the moving interface. Recently, atom probe tomography (APT) has revealed the actual state of alloying elements in view of the solute drag effect using B bearing Ti-IF steel [25,26]. Figure 7 shows that simultaneous addition of Ti and B retards the velocity of the interface movement more effectively than the addition of Ti or B alone [26]. Besides the fact that the simultaneous addition of Ti and B significantly suppresses the recovery and nucleation of recrystallization [26], it also retards the interface velocity of nuclei that grow into the deformed matrix. However, the underlying mechanism has not yet been elucidated. Therefore, it is necessary to clarify the mechanism by which the addition of Ti and/or B affects the growth of the recrystallized nuclei. The Ti-IF steel was used for the APT observations with constant 0.03% excess of solute Ti and varied the amount of B ranging from 0 to 14 ppm. Particular attention was given to the interface which commonly had high angle misorientations. Figure 8 reveals that co-segregation of Ti and B occurred and that there was no precipitation of Ti-B at the interface of 0.0018C-0.051Ti-0.0014B steel [25]. In contrast there was only slight segregation of Ti into the interface of 0.0023C-0.052Ti-0.0001B steel [25]. Moreover, it was confirmed that the amount of Ti segregation increases as the amount of B increases, which implies that B and Ti have a tendency to co-segregate. The co-segregation of B and Ti was also verified by the first principle calculation for the symmetrical tilt boundary of α-Fe(111)Σ3 $\left[1\overset{ˉ}{1}0\right]$[26]. The first principle calculation has also been conducted for the interaction between B and other substitutional transition metals in an α matrix. As shown in Figure 9, in addition to Ti and Nb, Mn and Cu are predicted to have a relatively large attractive interaction with B in an α matrix [27]. This should be verified in future works.

### 2.4. Competing α recrystallization and α→γ reverse phase transformation during heating

**Figure 10.**
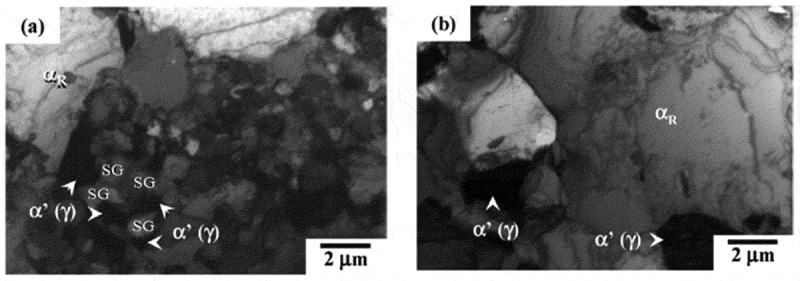
TEM micrographs showing (a) retardation of α recrystallization by the presence of finely transformed γ during intercritical annealing of 0.1C-2.0Mn steel and (b) α recrystallization restarting due to the Ostwald ripening of γ by further intercritical annealing [28]. SG: subgrain. With permission of ISIJ. Reproduced by permission from [28], copyright [2010, ISIJ]

In the process of producing high strength steel sheets consisting of α and αʹ through cold rolling and subsequent intercritical annealing, both α recrystallization and the α→γ reverse phase transformation take place in a competitive manner. Ogawa et al. [28] explored the microstructural evolution of bainitic steels (0.1C-2.0Mn) during heating after cold-rolling by 67%. During intercritical annealing at 750°C, recrystallization proceeds with time; however, it becomes stagnant during annealing between 100 s and 1000 s. It is believed that this is due to the presence of finely dispersed transformed γ at the subgrain boundaries in deformed α, which severely retards α recrystallization (Figure 10(a)). After it is held for 1000 s, reversely transformed γ grows due to Ostwald ripening (Figure 10(b)), which makes it possible for recrystallization to restart. Finally, a duplex microstructure comprising of α and γ (αʹ after cooling) is formed.

**Figure 11.**
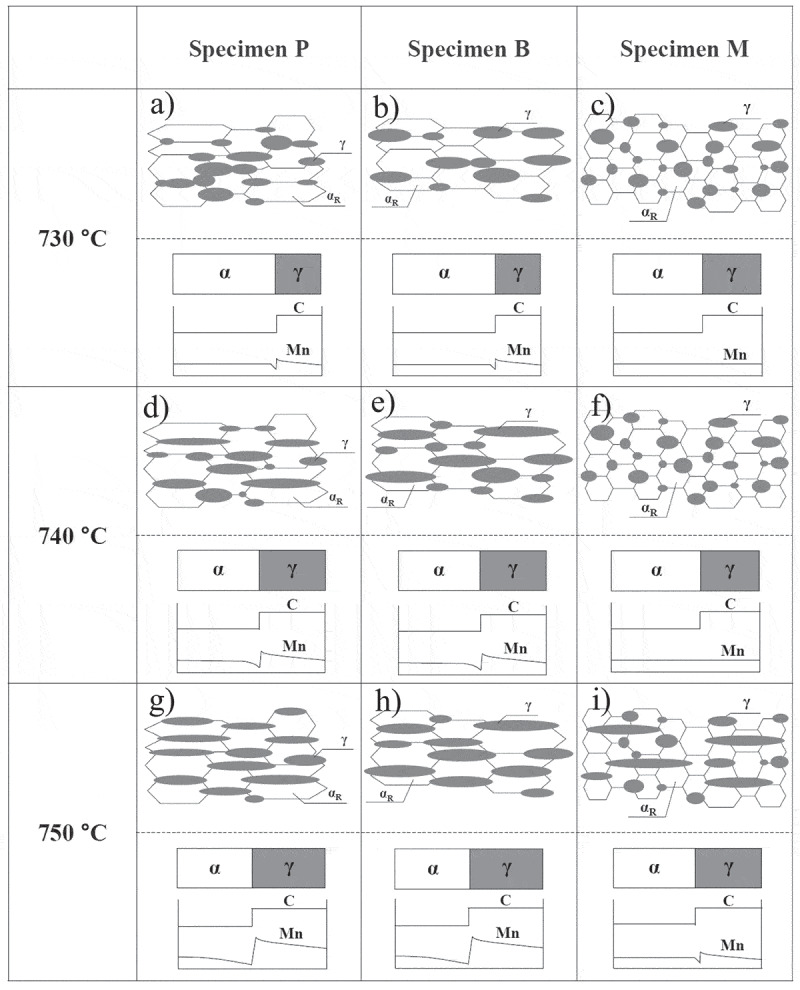
Schematics showing the microstructural evolution and distribution of C and Mn during heating from 730 to 750°C for specimens with initial pearlite (P), bainite (B), and martensite (M) structures before cold-rolling of 0.1C-2.0Mn steel [29]. With permission of Japan Inst. Met. Mater. Reproduced by permission from [29], copyright [2019, the Japan Inst. Met. Mater.]

Dannoshita et al. [29] investigated the competitive reaction between α recrystallization and the α→γ reverse phase transformation during heating by changing the initial microstructure prior to cold rolling (including pearlite, bainite, and martensite) in a 67% cold-rolled steel sheet of 0.1C-2.0Mn. As illustrated in Figure 11, the initial martensitic structure promoted α recrystallization, leading to the equi-axed fine α recrystallized grains and fine γ (αʹ) grains with a large volume fraction that were nucleated at the α recrystallized grain boundaries (Figure 11(c,f,i)). Alternatively, when the initial structure was pearlite or bainite, a substantial retardation of α recrystallization led to a competitive progression with α→γ reverse phase transformation. This resulted in a relatively coarse microstructure consisting of elongated α and γ (αʹ) structures (Figure 11(a,b,d,e,g,h)).

When 0.02% and 0.05%Nb is added to such a steel, three phenomena occur simultaneously: α recrystallization, α→γ reverse phase transformation, and NbC precipitation [30]. First, the formation of NbC clusters occurs during heating, which significantly retards the recovery and recrystallization of α. Consequently, α→γ reverse phase transformations occur at the unrecrystallized α subgrain boundaries, which leads to the formation of a very fine reversely transformed γ. As a result, α recrystallization is further suppressed by the presence of fine γ, finally leading to the formation of very fine α and γ (αʹ) duplex structures.

### 2.5. Evaluation of dislocation behavior during recovery and recrystallization by rigorous X-ray line profile analysis

**Figure 12.**
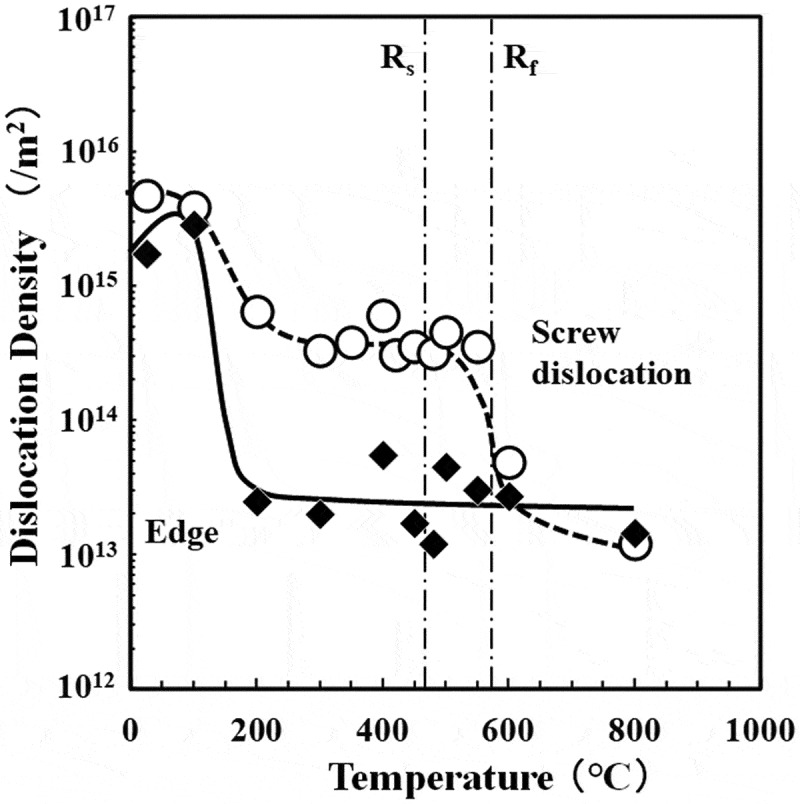
Changes in the dislocation densities of the edge and screw components as a function of annealing temperature for cold-rolled Fe-0.3Si alloy with 99.8% reduction [32]. (R_s_: Start of recrystallization, R_f_: Finish of recrystallization) With permission of ISIJ. Reproduced by permission from [32], copyright [2017, ISIJ]

Rigorous X-ray line profile analysis has made it possible to evaluate the dislocation density, the fraction of screw/edge dislocation components, and the degree of dislocation rearrangement [31]. The application of this method is exemplified by the dislocation behavior during the annealing of severely cold-rolled pure Fe, Fe-0.3Si, and Fe-0.3Al alloys [32]. Severely cold-rolled steel is known to exhibit unusual recrystallization behavior in the sense that deformed grains with α-fiber texture (RD//<011>), which are generally very difficult to recrystallize, create new recrystallized grains leading to the development of {100}<012> and {411}<148> textures [32]. Moreover, the addition of Si increases the density of the edge dislocations after cold rolling in comparison with pure Fe [32]. This is believed to be a result of the suppression of the cross slip of dislocations by the addition of Si. In the alloy Fe-0.3Si, the density of the edge dislocations decreases at temperatures as low as 200°C during heating (Figure 12). It is speculated that the annihilation of these edge dislocations occurs as the climbing up motion of edge dislocations takes place by absorbing vacancies at low temperatures. Once the temperature reaches 400°C, there is a significant decrease in the screw dislocation density and recrystallization progresses prominently (Figure 12). In contrast, in pure Fe, the density of the screw dislocations decreases at lower temperatures up to approximately 300°C and recrystallization commences at this temperature. Therefore, it is postulated that the addition of Si not only suppresses the cross-slip frequency of dislocations during cold rolling but also retards recovery and recrystallization during annealing due to the interaction between Si atoms and the dislocations. Furthermore, the addition of Al has similar effect as Si; however, the effect is smaller. It is essential to understand the role of alloying elements in the deformation and restoration process, and this requires further detailed research that makes effective use of cutting-edge analytical techniques and computational materials science.

## 3. Research into deformation and recrystallization exploiting advanced computational materials science

### 3.1. Prediction of heterogeneous deformation structure

There have been many studies on the prediction of the heterogeneous deformation structure and texture development of poly-crystal materials when they are plastically deformed. However, these predictions are not easy since the deformation is not only affected by the presence of neighboring grains, but also by the presence of a hard second phase. This is because the neighboring grains and hard second phase constrain the deformation of a particular grain. Furthermore, the addition of solute elements affects the slip behavior. In recent years, various approaches have been tested including crystal plasticity (CP)-finite element method (FEM) (CP-FEM) [33], CP-fast Fourier transform (CP-FFT) [34]. The latter is particularly advantageous due to its comparatively shorter calculation time.

Yamanaka [35] simulated the evolution of poly-crystalline γ structure of low C steel during hot compression test using CP-FFT. An initial three–dimensional (3D) γ structure composed of 200 grains was constructed using the phase field (PF) method. The size of the representative volume element was 12.8 × 12.8 × 12.8 μm^3^. The heterogeneous spatial distribution of the stored energy and the misorientation were obtained as a function of the applied strain after the material was deformed to relatively low strain under plane strain conditions. The results predict that the stored energy is heterogeneous and is locally high at certain grain boundaries and that there is large misorientation within a grain. This is an important result in terms of reproducing the three dimensionally heterogeneous deformation structures; therefore, it is expected to have many applications. Yamanaka [35] used the results for the γ deformation structure to predict the recrystallization of γ assuming classical nucleation theory and subsequent γ to α phase transformations via the PF model. However, further intensive work is required in order to predict the heterogeneous deformation structure with greater accuracy and even after large strain deformations. Moreover, the model should be verified using novel experimental techniques. However, the experimental justification for heterogeneous deformation structures is not practically easy because non-destructive 3D data on the dislocation density and misorientation with a sub-micrometer spatial accuracy are required, which would be likely enabled by exploiting focused pencil beam 3D-XRD [36,37].

### 3.2. Coupled analysis of plastic deformation and recrystallization/phase transformation

**Figure 13.**
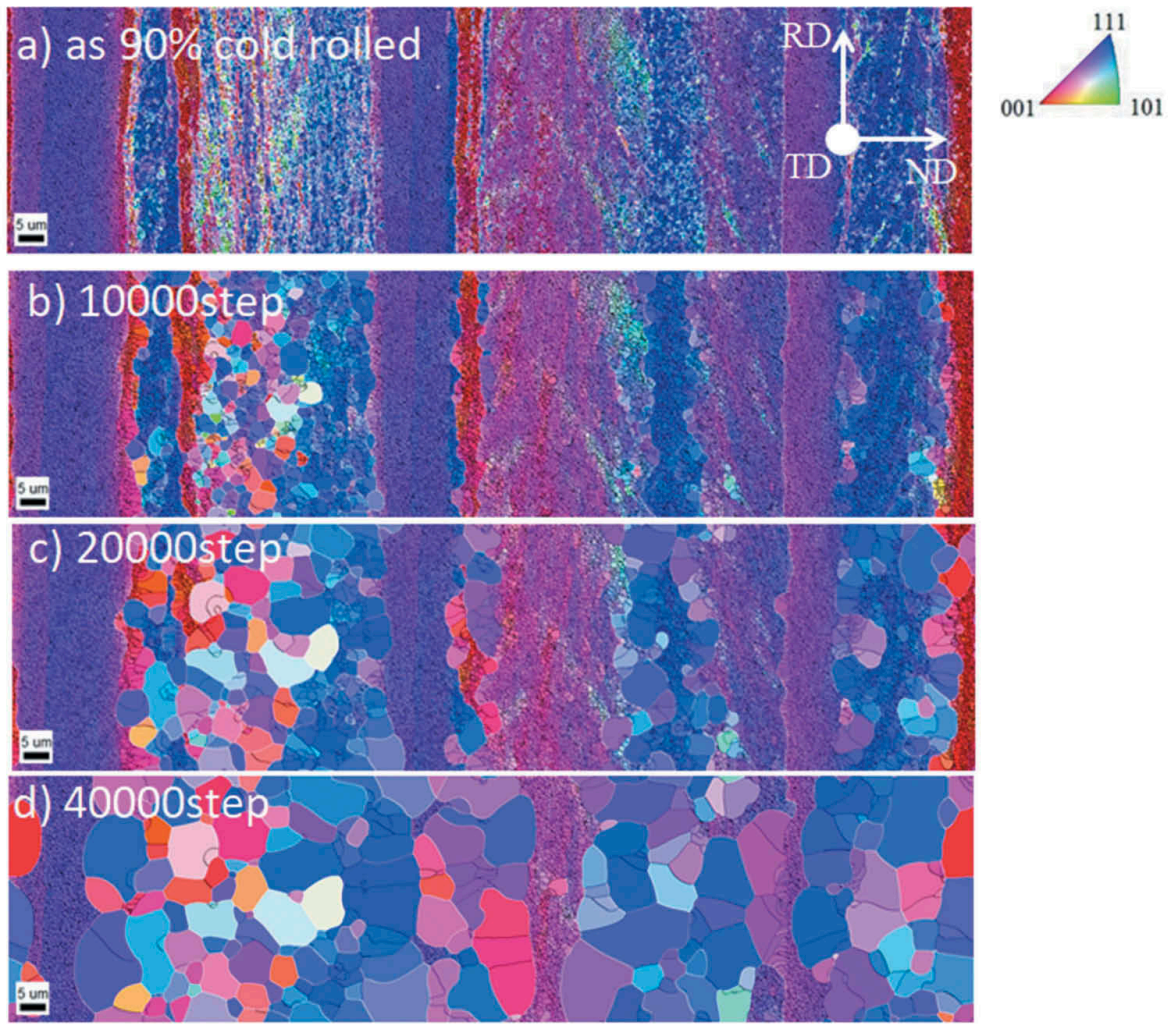
Simulated microstructural evolution of 90% cold-rolled Fe during recrystallization [40]. (a) ND orientation map of the converted subgrain structure from EBSD data of cold-rolled sheet, and partially recrystallized structures with time steps of (b) 10000, (c) 20000, and (d) 40000. With permission of ISIJ. Reproduced by permission from [40], copyright [2019, ISIJ]

**Figure 14.**
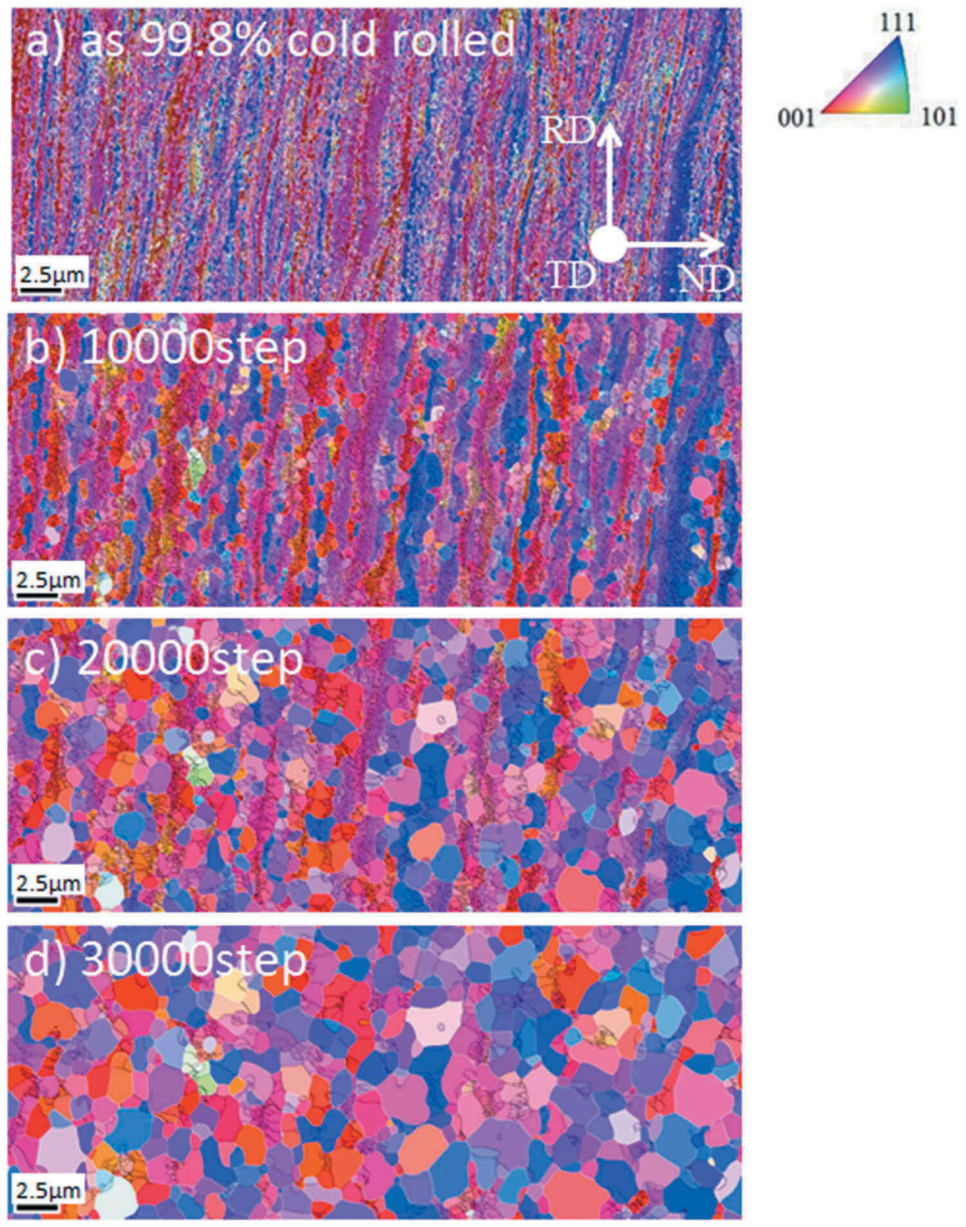
Simulated microstructural evolution of 99.8% cold-rolled Fe during recrystallization [40]. (a) ND orientation map of converted subgrain structure from EBSD data of cold-rolled sheet, and partially recrystallized structures with time steps of (b) 10000, (c) 20000 and (d) 30000. With permission of ISIJ. Reproduced by permission from [40], copyright [2019, ISIJ]

**Figure 15.**
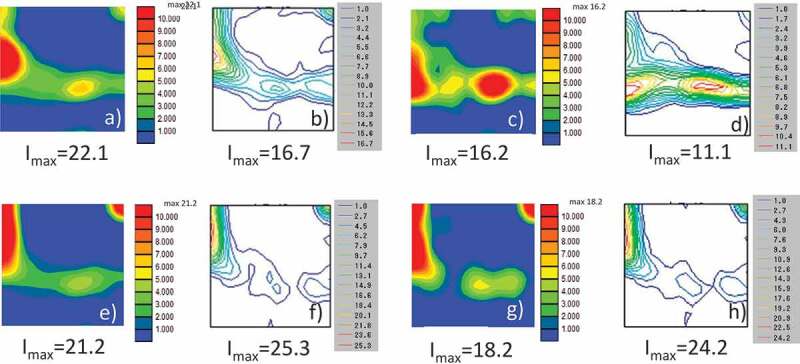
φ_2_ = 45° sections of ODFs (Orientation Distribution Functions): (a) converted [40] and (b) experimentally determined [41] 90% cold-rolled Fe. (c) Simulated [40] and (d) experimentally determined [41] 90% cold-rolled and subsequently recrystallized Fe. (e) Converted [40] and (f) experimentally determined 99.8% cold-rolled Fe [41]. (g) Simulated [40] and (h) experimentally determined 99.8% cold-rolled and subsequently recrystallized Fe [41]

Heterogeneous deformation structures are important as they act as preferential nucleation sites for recrystallization and phase transformation. Research attempting to construct a reliable model that can predict the microstructure and texture evolution from upstream to downstream has been advanced by the coupling model of deformation and recrystallization/phase transformation [33,35,38,39]. For instance, Takaki et al. reported a two-dimensional (2D) model of the static recrystallization of a face centered-cubic (fcc) metal by coupling CP-FEM for deformation and PF for recrystallization [38]. Yamanaka et al. reported a 3D model of the evolution of the γ structure during the hot compression of low C steel by coupling 3D CP-FFT for deformation and PF for recrystallization and phase transformation [35,39]. As far as the nucleation process of recrystallization is concerned, these models assumed that nucleation occurs preferentially in areas where the stored energy exceeds a certain value. In contrast, Suwa et al. conducted a 3D PF simulation of primary recrystallization, assuming that the nucleation of primary recrystallized grains is a type of abnormal subgrain growth process [40]. First, the initial subgrain structure was constructed using EBSD data for 90% and 99.8% severely cold-rolled pure Fe (Figures 13(a) and 14(a)). The simulated results for recrystallization qualitatively reproduced the experimental ones in the 90% case in that recrystallization occurred in a pattern of nucleation and growth, and that the ND//<111> recrystallization texture developed (Figures 13 and 15(c,d)) [41]. When the cold-rolling reduction of pure Fe increased from 90% to 99.8%, continuous recrystallization-like behavior was also recognized by the PF simulation which reproduces the experimental results (Figures 14 and 15(g,h)). The simulated results for the 99.8% cold-rolling reduction revealed that the cold-rolling texture remains unchanged even when recrystallization is complete (Figure 15(e,g)), which is in accordance with the experimental results (Figure 15(f,h)). Concerning the development of recrystallization texture after heavy cold-rolling, it has been reported that ~{411}<148> texture develops in Fe- 0.3Si alloy [42], ultra-low C steel [43] and Fe-3.2Si alloy [44]; however, this tendency is different from that of pure Fe [41] and Fe-0.3Al alloy [42]. This implies that alloying elements significantly affect recrystallization behavior and texture formation. Application of simulation model to these unsolved problems in connection with systematic experiments is considered future important subjects. Furthermore, in order to establish a more accurate simulation methodology for rolling and recrystallization, the following areas must be addressed: (1) a suitable method must be used to reconstruct the subgrain structure from the deformation structure and (2) an accurate database must be developed for grain boundary energy and grain boundary mobility as a function of the grain boundary characteristics. In contrast, for the simulated phase transformation, a variant selection rule during γ to α phase transformations should be established. For instance, Furuhara et al. proposed a variant selection rule for when bainite transformations occur [45]. A deeper understanding of interface energy/interface mobility, variant selection and their database are required for more accurate predictions of the microstructure and texture during phase transformation.

### 3.3. Analysis of the interaction between dislocation and solute atoms using first principle calculations and molecular dynamics

In order to understand the evolution of heterogeneous deformation structures, it is necessary to know how solute atoms affect dislocation glide behavior. Waketa et al. systematically investigated the interaction between screw dislocation core and various substitutional atoms using first principle calculations combined with molecular dynamics [46]. They found that: (1) many solute atoms have an attractive interaction with the screw dislocation core; (2) some solutes such as Si, P, and Cu reduce the Peierls potential needed for the screw dislocation to move; and (3) the mechanism by which solutes affect dislocation glide is attributed to the electronic behavior (chemical misfit).

The interaction between the dislocation core and solute atoms not only affects the dislocation glide in the early stage of deformation, but also has a significant effect on the collective dislocation motion related to work hardening and dynamic recovery in the later stages of deformation and subsequent plastic instability. Based on this, we anticipate the development of a multi-scale approach that relates atomic-scale calculations to macro-scale properties.

## 4. Future developments

The recent development of advanced analytical techniques is remarkable, and it is now possible to clearly observe phenomena that were previously invisible. Furthermore, the progress in computational materials science has enabled us to conduct multi-scale calculations and to understand phenomena at a level of detail that used to be un-calculable and speculative. The accurate prediction of phenomena, which cannot be tested experimentally, is also becoming possible and has various implications.

However, many problems remain outstanding:

1. The understanding and prediction of heterogeneous deformation structures, both experimentally and theoretically, are still insufficient and further advancement is required.
2. The mechanism underlying orientation selection during recrystallization following deformation must be clarified, especially focusing on the recovery and nucleation of recrystallization.
3. The effect of alloying elements on the dislocation core structure must be fundamentally elucidated in conjunction with the movement of single dislocations and the collective movement of dislocations.
4. The interface energy and mobility should be systematically elucidated together with the effect of alloying elements while taking into consideration the interface character.
5. Finally, advanced analytical techniques should be combined with computational materials science for further materials research in order to solve crucial technical problems, and to realize the profound capabilities of materials.
